# Brief data report on prototype of moral personality and environmentalism

**DOI:** 10.1016/j.dib.2017.10.013

**Published:** 2017-10-09

**Authors:** Fanli Jia

**Affiliations:** Department of Psychology, Seton Hall University, United States

## Abstract

The data presented here was partially published in the article “Are Environmental Issues Moral Issues? Moral Identity in Relation to Protecting the Natural World” (Jia et al., 2017) [1]. The data was collected at State University of New York at Oneonta in 2016. It included a self-report questionnaire of moral identity, generativity, community engagement, environmental involvement, environmental identity, and demographic information.

**Specifications Table**

**Table:** 

Subject area	Psychology
More specific subject area	Environmental and Moral Psychology
Type of data	Quantitative data
How data was acquired	Survey, Excel
Data format	Raw data
Experimental factors	Correlational design. Individual clusters of moral identity was computed; high (environmental activists) vs. low (environmental non-activists) environmental involvement was computed by z-scores.
Experimental features	13 domains of moral identity, environmental activists vs. non-activists
Data source location	State University of New York at Oneonta
Data accessibility	Data is within the article

**Value of the data**

• The raw data of self-reported prototypes of moral personality (moral identity, generativity, community engagement) and environmentalism (environmental involvement and environmental identity). The data set may be examined using such statistical methods as analysis of variance, regression, factor analysis, cluster analysis, or structural equation modeling.
• The dataset includes demographic attributes. The categorical coding of these fields may allow for comparisons of different groups of SES, age and culture of origin to parallel samples in other similar studies elsewhere.
• The self-reported moral identity are defined in three steps: 1) free-listing moral attributes; 2) rating and selecting moral values; 3) categorizing moral identity within 13 personality domains. Analyses resulting from data generated by moral identities may be compared to findings from other datasets collected using alternative methodological models of moral identity.

## 1 Data

The date set contains self-reported responses of 164 participants. The data is grouped into fields that recorded moral identity, generativity, community engagement, environmental identity and environmental involvement. Table 1 summarizes the variables contained in the provided data file.

**Table 1:** Data files.

Variable	Variable description	Variable type	Variable labels
Culture	Culture	Nominal	4 = Americans
Gender	Gender	Nominal	1 = Male; 2 = Female
BirthYear	Year of Birth (YYYY, e.g. 1991)	String	Text
Birthmonth	Month of Birth	Nominal	1 = January … 12 = December
BirthCountry1	Country of Birth	Nominal	1 = USA; 2 = outside USA
BirthCountry2	Country of Birth	String	Text
LivingUSA	If you were not born in USA: For how many years have you been living in USA?	String	Text
Ethnicity	What are your ethnic or cultural origins? (e.g., American, British, French, Chinese, Italian…)	String	Text
FatherOcc	What is your father's current occupation?	String	Text
FatherEdu	Choose the highest level of education your father has attained.	Numeric	1 = Some high school studies;
			2 = completed high school;
			3 = some college and university studies;\
			4 = completed college diploma;
			5 = completed undergraduate degree;
			6 = some postgraduate studies;
			7 = completed graduate or professional degree
MotherOcc	What is your mother's current occupation?	String	Text
MotherEdu	Choose the highest level of education your mother has attained.	Numeric	1 = Some high school studies;
			2 = completed high school;
			3 = some college and university studies;
			4 = completed college diploma;
			5 = completed undergraduate degree; 6 = some postgraduate studies;
			7 = completed graduate or professional degree
Free Listing	Please write down 5 attributes describing a highly moral person in your personal view.	String	Text
Rating_accepting… Rating_ambitious a	Describes a highly moral person-accepting …	Numeric	1 = Not at all; 2 = a little bit; 3 = somewhat; 4 = fairly well; 5 = Extremely well
	Describes a highly moral person-ambitious		
Selected_accepting …	In the next step please select 12–15 qualities that define the core of a highly moral person from…	Nominal	1 = selected
Selected_ambitious a			
LGS1 …	Generative Concerns	Scale	As reported
LGS20 b			
CE1…	Community Engagement	Scale	As reported
CE30^c^			
EI1 …	Environmental Identity	Scale	As reported
EI12^d^			
Ein1 …	Environmental Involvement	Scale	As reported
Ein11^e^			

a For 90 moral values; refer to supplementary material.

b LGS: 20 generative items, refer to supplementary material.

c CE 30 community engagement items, refer to supplementary material.

d EI: 12 environmental identity items, refer to supplementary material.

e Ein: 11 environmental involvement items, refer to supplementary material.

Descriptive statistics on demographic variables can be obtained from functions in Excel file. In addition, users could transform the format of the data file to Statistical Package for the Social Sciences (SPSS), a statistical software that is often used in Social Science research. Syntax of descriptive analysis for demographic variables is provided:

Mean and Standard Deviation:

DESCRIPTIVES VARIABLES=Culture BirthYear birthmonth BirthCountry1 FatherEdu MotherEdu.

/STATISTICS=MEAN STDDEV.

Frequency:

FREQUENCIES VARIABLES=gender BirthCountry2 LivingUSA.

/ORDER=ANALYSIS.

## 2 Design

The dataset contained in the Excel file includes demographic information and self-reported responses to moral identity, generativity, community engagement, environmental involvement, and environmental identity, respectively. The data is based on correlational design.

Moral identity questionnaire includes moral attributes which individuals used to define their personal moral identity. This measure has been used in recent studies [1], [2], [3]. A total of 13 moral domains that make up the list of 90 value attributes was used in the present data set (for a full list of all value attributes and their categorizations, see Jia et al. [1] in Table 1). In order to identify prototypes of moral personality, the moral identity questionnaire could be used in two ways: first, participants would rate each attribute to define a highly moral person; second, participants would select 12 to 15 attributes according to their own personal view that defined “the core of a highly moral person”. The remainder of the questionnaire will be based on those 12–15 values that individuals had selected for themselves to define a highly moral person.

The Environmental Involvement Scale includes 11 items measure [4] to capture additional aspects of environmental engagement. In order to determine environmental activists and non-activists, a cut-off criterion may be applied to identify the two groups: participants who scored over 1.5 standard deviations above the average score on the Environmental Involvement questionnaire were considered as environmental activists; participants who scored over 1.5 standard deviations below the average score of the Environmental Involvement questionnaire were considered as non-activists. This procedure has been used in previous studies [4], [5] to create, and then compare and contrast sub-groups of participants on a particular domain.

## 3 Materials

The Word file included in the supplementary material contains the full phrasing of the survey items.

Demographic information included participants’ age, culture of origin, father/mother's occupations, ethnicity, and length of residency in USA.

The questionnaire packet included three sub-measures of moral identity, generativity, community engagement, environmental identity and environmental involvement. Reliability scores and validity checks were reported in the published articles [1].

## 4 Methods

Participants (*n* = 164) were recruited from Introduction to Psychology classes at State University of New York, Oneonta. A majority of the participants were of European American background (e.g., English, Italian, Polish, German, French etc.). Anonymity was guaranteed and participants received course credits for attending the session.

All participants were asked to fill out a questionnaire packet. Moral identity were measured using a questionnaire version of the moral identity interview (see design section). Participants were asked to free-list at least five moral attributes to describe a highly moral person. Then researchers presented a list of 90 moral attributes (e.g., dependable, caring, tolerant) describing a highly moral person. They were instructed to select 12–15 attributes that according to their own personal view, defined “the core of a highly moral person”. All 90 attributes were classified into domains of benevolence-dependability, benevolence-caring; universality-tolerance, universality-concern; conformity-interpersonal, conformity-rule; self-direction; and achievement, by five independent coders. How often participants chose a particular value domain was counted. These scores were divided by the overall number of attributes they identified and multiplied by 100, yielding a percentage score of the relative importance of each value domain for defining a person's moral identity.

Two questionnaires were used to assess the related but distinct environmental components of involvement, and identity. The Environmental Involvement Scale consists of 11 items that assessed a range of environmental actions. Participants rated, on a 5-point Likert-type scale from 0 (never) to 4 (a lot), the frequency with which they engaged in each of the 11 activities such as ‘‘Bought rechargeable batteries’’ and ‘‘Signed a petition or participated in a campaign to make policy or actions more environmentally friendly’’ within the last year. The Environmental Identity Scale, developed by Clayton [6] was used to measure the strength of an identity. It includes 12 items rated on a Likert-type scale from 1 (not true of me at all) to 7 (completely true of me). ‘‘I feel that I receive spiritual sustenance from experiences with nature’’ is an example of an item.

The Generativity Scale [7] is a 20-item self-report questionnaire that measures generative concerns using a 9-point scale, ranging from 1 (very strongly disagree) to 9 (very strongly agree). A sample item would be ‘‘I try to pass along the knowledge I have gained through my experiences.’’ Responses to items were summed together into a total score.

The Community Engagement scale [8] includes 30 activities related to prosocial (e.g., “Visited or helped out people who were sick”, community;“Helped organize neighborhood or community events"), and political involvement (e.g., “Worked on a political campaign”). Participants rated the frequency with which they engaged in each of these activities within the last year, using a 5-point Likert scale from 0 (you never did this) to 4 (you did this a lot).

## Funding

This research was supported by the Seton Hall University Research Council.
