# Supplementary material for: Brief data report on prototype of moral personality and environmentalism
Source: Data Brief. 2017 Oct 9;15:540–4. doi: 10.1016/j.dib.2017.10.013 (PMC5651487; doi:10.1016/j.dib.2017.10.013)
Supplement: Supplementary file 1 — Supplementary material [file mmc1.docx]

There is no conflict of interest.
