# Supplementary material for: Brief data report on prototype of moral personality and environmentalism
Source: Data Brief. 2017 Oct 9;15:540–4. doi: 10.1016/j.dib.2017.10.013 (PMC5651487; doi:10.1016/j.dib.2017.10.013)
Supplement: Supplementary file 2 — Supplementary material [file mmc2.docx]

**Q1: What describes a highly moral person part I: Free listing**

Please write down 5 attributes describing a highly moral person in your personal view.

|  |  |  |  |  |
| --- | --- | --- | --- | --- |

**Q2: What describes a highly moral person part II: Rating**

Please rate each attribute according to how well it describes a highly moral person on a

5-­‐point scale.

1 = describes a highly moral person not at all

2 = describes a highly moral person a little bit

3 = describes a highly moral person somewhat

4 = describes a highly moral person fairly well

5 = describes a highly moral person extremely well

| _____ strong | _____ proper | _____ courageous | _____ ambitious |
| --- | --- | --- | --- |
| _____ honest | _____ incorruptible | _____ solidaric | _____ caring |
| _____ credible | _____ ethical | _____ sociable | _____ clean |
| _____ obedient | _____ hardworking | _____ selfless | _____ compassionate |
| _____ peaceful | _____ reliable | _____ conscientious | _____ educated |
| _____ forgiving | _____ helpful | _____ understanding | _____ follows the rules |
| _____ perseveres | _____ modest | _____ friendly | _____ fun |
| _____ consistent | _____ optimistic | _____ knows what is right/wrong | _____ intelligent |
| _____ fair | _____ patient | _____ prudent | _____ knowledgeable |
| _____ cooperative | _____ diligent | _____ generous | _____ law-abiding |
| _____ truthful | _____ benevolent | _____ motivated | _____ loving |
| _____ patriotic | _____ thrifty | _____ honourable | _____ loyal |
| _____active | _____ warm-hearted | _____ principled | _____ proud |
| _____ sharing | _____exemplary | _____ civilized | _____ religious |
| _____ non-judgmental | _____ outgoing | _____ kind | _____ respectful |
| _____ independent | _____ wise | _____ considerate | _____ responsible |
| _____ genuine | _____ rational | _____ dependable | _____ righteous |
| _____ tolerant | _____ dedicated | _____ open-minded | _____ self-confident |
| _____ good | _____ humble | _____ careful | _____ self-disciplined |
| _____ healthy | _____ faithful | _____ accepting | _____ sincere |
| _____ has high standards | _____ listens | _____ happy | _____ trustworthy |
| _____ grateful | _____ courteous | _____ altruistic | _____ upstanding |
| _____ just | _____ makes the right choices | _____ filial piety |  |

**Q3: What describes a highly moral person part III: Selecting**

Please select 12 attributes according to how well it describes a highly moral person in your personal view.

|  |  |  |  |  |  |
| --- | --- | --- | --- | --- | --- |
|  |  |  |  |  |  |

**Q4: Relationships with Others (Generativity)**

Use the following scale to indicate your agreement or disagreement with each statement about your relationships with others.

| **1** | **2** | **3** | **4** | **5** | **6** | **7** | **8** | **9** |
| --- | --- | --- | --- | --- | --- | --- | --- | --- |
| very strongly disagree | strongly disagree | moderately disagree | slightly disagree | neither agree nor disagree | slightly agree | moderately agree | strongly agree | very strongly agree |

|  | 1. I try to pass along the knowledge I have gained through my experiences. |
| --- | --- |
|  | 2. I do not feel that other people need me. |
|  | 3. I think I would like the work of a teacher. |
|  | 4. I feel as though I have made a difference to many people. |
|  | 5. I do not volunteer to work for a charity. |
|  | 6. I have made and created things that have had an impact on other people. |
|  | 7. I try to be creative in most things that I do. |
|  | 8. I think that I will be remembered for a long time after I die. |
|  | 9. I believe that society cannot be responsible for providing food and shelter for all homeless people. |
|  | 10. Others would say that I have made unique contributions to society. |
|  | 11. If I were unable to have children, I would like to adopt children. |
|  | 12. I have important skills that I try to teach others. |
|  | 13. I feel that I have done nothing that will survive after I die. |
|  | 14. In general, my actions do not have a positive effect on others. |
|  | 15. I feel as though I have done nothing of worth to contribute to others. |
|  | 16. I have made many commitments to many different kinds of people, groups, and activities in my life. |
|  | 17. Other people say that I am a very productive person. |
|  | 18. I have a responsibility to improve the neighborhood in which I live. |
|  | 19. People come to me for advice. |
|  | 20. I feel as though my contributions will exist after I die. |

**Q5: Community Engagement**

The following is a list of community and political activities that people can get involved in. For each of these activities, please use the following scale to indicate whether, in the **last year** you did this:

| **0** | **1** | **2** | **3** | **4** |
| --- | --- | --- | --- | --- |
| never | once or twice | a few times | a fair bit | a lot |

|  | 1. Visited or helped out people who were sick |  | 21. Gave help (ex: money, food, clothing, rides) to friends or neighbours who need it |
| --- | --- | --- | --- |
|  | 2. Took care of other families’ children (on an unpaid basis) |  | 22. Served as a member of an organizing committee or board for a club or organization. |
|  | 3. Participated in a church-connected group |  | 23. Wrote a letter to a community newspaper or publication |
|  | 4. Joined in a protest march, meeting, or demonstration |  | 24. Led or helped out with a children’s group or club |
|  | 5. Participated in an ethnic club or organization |  | 25. Attended a demonstration |
|  | 6. Participated in a social or cultural group or organization (ex: a choir) |  | 26. Collected signatures for a petition drive |
|  | 7. Helped with a fund-raising project |  | 27. Contributed time or money to an environmental or wildlife conservation group |
|  | 8. Participated in a sports team or club |  | 28. Gave money to a cause |
|  | 9. Signed a petition |  | 29. Got information about community activities from a local community information centre. |
|  | 10. Participated in a political party, club or organization |  | 30. Volunteered at a school event or function |
|  | 11. Helped organize neighborhood or community events (ex: carnivals, potluck dinners etc.) |  |  |
|  | 12. Helped prepare and make verbal and written presentations to organizations, agencies, conferences or politicians. |  |  |
|  | 13. Did things to help improve your neighborhood (ex: helped clean it up) |  |  |
|  | 14. Ran for a position in government |  |  |
|  | 15. Participated in a discussion about a social or political issue |  |  |
|  | 16. Volunteered with a community service organization |  |  |
|  | 17. Contacted a public official by phone or mail to tell him/her how you felt about a particular issue. |  |  |

**Q6: Thoughts about Nature (Environmental Identity)**

Please indicate the extent to which each of the following statements describes you by using the appropriate number from the scale below.

| **1** | **2** | **3** | **4** | **5** | **6** | **7** |
| --- | --- | --- | --- | --- | --- | --- |
| not at all  true of me |  |  | neither true  nor untrue |  |  | completely  true of me |

|  | 1. I spend a lot of time in natural settings (woods, mountains, desert, lakes, ocean). |
| --- | --- |
|  | 2. I think of myself as a part of nature, not separate from it. |
|  | 3. If I had enough time or money, I would certainly devote some of it to working to protect the environment. |
|  | 4. When I am upset or stressed, I can feel better by spending some time outdoors "communing with nature". |
|  | 5. I feel that I have a lot in common with other species. |
|  | 6. Behaving responsibly toward the earth -- living a sustainable lifestyle -- is part of my moral code. |
|  | 7. Learning about the natural world should be an important part of every child's upbringing. |
|  | 8. I would rather live in a small room or house with a nice view than a bigger room or house with a view of other buildings. |
|  | 9. I would feel that an important part of my life was missing if I was not able to get out and enjoy nature from time to time. |
|  | 10. I have never seen a work of art that is as beautiful as a work of nature, like a sunset or a mountain range. |
|  | 11. I feel that I receive spiritual sustenance from experiences with nature. |
|  | 12. I keep mementos from the outdoors in my room, like shells or rocks or feathers |

**Q7: Environmental Involvement**

The following is a list of environmental activities that people can get involved in. For each of these activities, please use the following scale to indicate whether, in the **last year** you did this:

| **0** | **1** | **2** | **3** | **4** |
| --- | --- | --- | --- | --- |
| never | once or twice | a few times | a fair bit | a lot |

|  | 1. Started buying a product because you think it protects the environment (ex., soap) |  | 5. Had a serious discussion on environmental issues with your parent(s)/child(ren) |
| --- | --- | --- | --- |
|  | 2. Read a conservation or environmental magazine |  | 6. Watched a television special on the environment |
|  | 3. Composted your kitchen waste. |  | 7. Taken steps to reduce energy use (ex., turn off water, lights) |
|  | 4. Contributed time or money to an environmental or wildlife conservation group |  | 8. Recycled newspapers, glass, or other items |
|  | 9. Buy rechargeable batteries. |  | 10. Buy second-hand goods. |
|  | 11. Signed a petition or participated in a campaign to make policy or actions more environmentally-friendly. |  |  |
